# Prevalence and Risk Factors of Pelvic Floor Disorders After Delivery in Japanese Women Using the Pelvic Floor Distress Inventory: A Retrospective Cohort Study

**DOI:** 10.7759/cureus.40152

**Published:** 2023-06-08

**Authors:** Tokumasa Suemitsu, Kazumi Mikuni, Hiroki Matsui, Makoto Suzuki, Tomoko Takahashi

**Affiliations:** 1 Obstetrics and Gynecology, Kameda Medical Center, Kamogawa, JPN; 2 Gastroenterological Surgery, Kameda Medical Center, Kamogawa, JPN; 3 Clinical Research Support Division, Kameda Institute for Health Science, Kameda College of Health Sciences, Kamogawa, JPN; 4 Health-Care Center, Tomishiro Central Hospital Health-Care Center, Tomigusuku, JPN

**Keywords:** urinary incontinence, prevalence, pelvic floor distress inventory-20 (pfdi-20), pelvic floor disorders, japanese postpartum woman, epidural delivery

## Abstract

Introduction

Female pelvic floor disorders (PFDs) include clinical conditions such as urinary and fecal incontinence and pelvic organ prolapse. Disease-specific questionnaires like the Pelvic Floor Distress Inventory-20 (PFDI-20) have facilitated pelvic floor disorder assessment. We aimed to investigate the prevalence of pelvic floor disorders in Japanese women after different modes of delivery and the association of pelvic floor disorders with epidural anesthesia.

Material and methods

We included 212 women who gave birth at our institution. The PFDI-20 questionnaire (validated in Japanese) was used to evaluate the symptoms of pelvic floor disorders 6-15 months postpartum.

Results

Out of the 212 postpartum women who participated in this study, 156 (73.6%) had symptoms of pelvic floor disorder; the most prevalent symptom was urinary distress inventory in 114 (53.8%) women [79 (37.3%) of them experienced urine leakage related to increased abdominal pressure]. A comparison of the epidural and non-epidural groups to determine the association between pelvic floor disorder and delivery mode revealed a higher disease burden score of 8.67 points in the epidural group.

Conclusion

The prevalence of pelvic floor disorder symptoms is relatively high, affecting 156 (73.6%) of 212 women. Accurate diagnosis of women and appropriate and regular follow-up until improvement of their symptoms are crucial. Furthermore, healthcare workers should advise pregnant women on whether to choose vaginal delivery with or without anesthesia. To the best of our knowledge, our study is the first to investigate postpartum pelvic floor disorder in Japan.

## Introduction

Female pelvic floor disorders (PFD) comprise various clinical conditions, such as urinary and fecal incontinence and pelvic organ prolapse. PFD assessment has been facilitated by the development of disease-specific questionnaires, such as the Pelvic Floor Distress Inventory-20 (PFDI-20). The PFDI-20 is a 20-item questionnaire divided into scores on three scales and evaluating the pelvic organ prolapse distress inventory (POPDI), colorectal anal distress inventory (CRADI), and urinary distress inventory (UDI) [1].

Pregnancy and labor are known risk factors for PFD [1]. Among the 196 surveyed Australian women, PFD was prevalent at six months postpartum in 97%, according to the PFDI-20 survey [2]. In a study conducted in Israel, 117 women were investigated during late pregnancy and puerperium. PFD assessment at three months postpartum showed POPDI in 12.8%, CRADI in 20.7%, and UDI in 15.8% of them. Despite these, mixed trends of spontaneous recovery following childbirth have been reported [3]. Additionally, there are mixed trends regarding the prevalence and recovery period. We hypothesized that symptoms of PFD would persist following the point at which the woman becomes more active (such as returning to her normal daily routine) or as the infant’s weight increases, compared to the early postpartum period, like three months after delivery. The prevalence of PFD after delivery and the recovery in Japanese women are unclear; thus, we investigated symptoms of PFD during the postpartum period, with a focus on those occurring later.

Anim-Somuah et al. reported that epidural anesthesia was associated with a prolonged second stage of labor [4]. Vaginal delivery with epidural anesthesia is associated with an increased risk for instrumental delivery [5]. Moreover, a prolonged second stage of labor and forceps delivery may injure pelvic floor muscles, causing PFD in postpartum women [6-7]. Epidural anesthesia is associated with urinary incontinence immediately after delivery but not at three and 12 months postpartum [8]. Therefore, the hypothesis that epidural anesthesia affects pelvic floor function is reasonable. In contrast, epidural anesthesia reportedly relaxes the pelvic floor muscles, reduces indirect and direct trauma to the pelvic floor, and prevents the development of symptoms related to pelvic floor trauma [9]. Therefore, the association of PFD risk with the use of epidural anesthesia in labor is controversial. Thus, we analyzed the data to reveal the association between the mode of delivery and the prevalence of PFD symptoms. However, data on the association of PFD prevalence with epidural anesthesia in postpartum Japanese women are scarce. Therefore, we conducted this study to further investigate this association and the prevalence of PFD symptoms in Japanese women six to fifteen months after delivery.

This article was previously presented as a meeting abstract at the 74th Annual Congress of the Japan Society of Obstetrics and Gynecology on August 5-7, 2022, and the 73rd Annual Congress of the Japan Society of Obstetrics and Gynecology on April 22-25 (Sunday) 2021, separately. This article was previously posted to the Research Square preprint server on June 1, 2022.

## Materials and methods

This was a retrospective cohort study of women who gave birth between July 2018 and May 2019 at the Kameda Medical Center, Chiba Prefecture, Japan, where an average of 600 deliveries are made annually.

All applicants were informed and enrolled by mail, which included an informed consent form. Hence, informed consent was received from all participants before the initiation of the study.

PFD was evaluated using the PFDI-20 questionnaire, which consists of 20 condition-specific measures of pelvic symptoms. It has three subscales: the Pelvic Organ Prolapse Distress Inventory with six items (POPDI-6), the Colorectal-Anal Distress Inventory with eight items (CRADI-8), and the Urinary Distress Inventory with six items (UDI-6).

Each question relates to the presence of an individual symptom. If the symptom is present, the responder scores it on a 4-point scale to indicate the extent of inconvenience the symptom elicits; a score of 1 is "not at all", and a score of 4 is "quite a bit."

POPDI-6, CRADI-8, and UDI-6 are each scored similarly. First, subscale scores for each scale are obtained by taking the mean value of all items answered within each subscale. These subscale scores are determined by multiplying each scale’s mean score by 25, which gives each subscale score a range of 0-100. As a result, adding up the subscale scores (which range from 0 to 300) yields the overall scale score. A high score indicates more symptoms and higher levels of inconvenience caused by the symptoms. The questionnaire was based on the validated Japanese version of the PFDI-20 [10]. The questionnaire was digitized as a web form without any modifications to the original form.

Participants were sent a questionnaire along with an explanation document about the PFDI-20 six to fifteen months after delivery. The target study population was 6-15 months postpartum in patients who gave birth at the Kameda Medical Center. The inclusion criteria for the study were non-preterm deliveries, singleton pregnancies, and fluency in Japanese. Individuals who either did not respond or did not respond well to the questionnaire were excluded from the analysis.

We divided all women into three groups: the cesarean section, epidural (vaginal delivery with epidural anesthesia), and non-epidural groups. Furthermore, in the cesarean-section group, emergency cesarean-section was divided into four groups: emergency cesarean-section, with epidural in labor, without epidural in labor, and not in labor. Epidural anesthesia was mainly indicated based on the patient’s demand and in cases where it was required because of medical issues (e.g., hypertensive disorders of pregnancy).

The PFDI-20 questionnaire was answered at 6-15 months postpartum, reflecting the respondent’s state during that period. A "yes" on the questionnaire represented a positive result, indicating the occurrence of PFD. Study identification numbers were assigned to each woman, and their medical information was obtained from the center’s medical records.

Statistical analyses

We obtained data on age, parity, maternal body weight before the pregnancy, mode of delivery, and the result of delivery mode. Continuous variables with a normal distribution are presented as mean and median values. We compared maternal, fetal, and neonatal characteristics with PFDI-20 (POPDI, CRADI, and UDI) in each group. Continuous variables were compared using the one-way ANOVA, and categorical variables were evaluated using the chi-squared test. A linear regression model was constructed for the PFDI-20 (POPDI, CRADI, and UDI), with adjustments for confounding factors (age, parity, and pre-pregnancy body weight). All confounding factors used were derived from the findings of previous studies. Causal-directed acyclic graphs (DAGs) were used to identify potential interplay among variables [11]. Supplementary Material Data 1 shows the DAGs used in this study.

Maternal age and pre-pregnancy body weight reportedly influence the mode of delivery due to maternal obstetrical complications [12-13]. Moreover, a previous mode of delivery affects the choice of delivery mode in future pregnancies; for example, the rate of vaginal birth after a cesarean procedure is reduced by approximately 10-30% in Japan [14]. Factors such as these influence the PFD [15]; therefore, we considered these factors to be confounding factors. Statistical significance was set at p<0.05, and all statistical analyses were conducted using R version 3.6.3 on July 22, 2011, at 23:54:00 (R Foundation for Statistical Computing, Vienna, Austria).

We assumed that any missing data was completely missing at random; hence, we conducted all analyses using the available data.

Ethics statement

This study was approved by the institutional ethical review board of our hospital on August 28th, 2019 (approval ID: 19-052).

## Results

**Figure 1:**
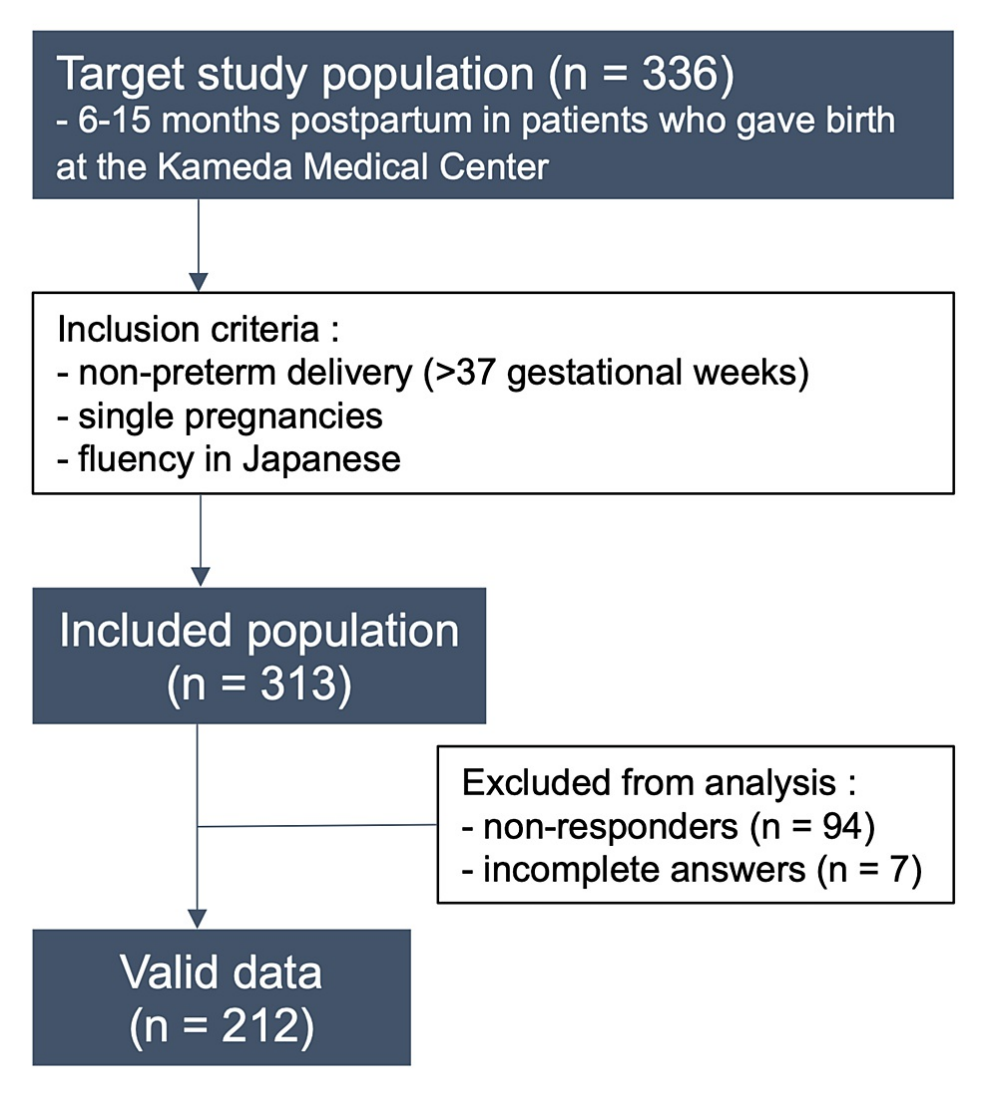
Study flow chart

Out of 336 women included in the study, 313 met the inclusion criteria. However, 101 women were excluded from the study due to no or incomplete responses. Ultimately, 212 postpartum women were analyzed (Figure 1).

Maternal, fetal, and neonatal characteristics (Table 1) revealed that the mode of delivery included emergency cesarean sections in 14 (6.6%) women, elective cesarean sections in 30 (14.2%), vaginal deliveries with epidural anesthesia in 21 (9.9%), and vaginal deliveries without epidural anesthesia in 147 (69.3%) women.

**Table 1:** Maternal, fetal, and neonatal characteristics of the study groups SD: standard deviation. ^†^The duration of labor in emergency cesarean section group in labor.

Characteristics	Overall n = 212	Cesarean section n = 44	Vaginal delivery with epidural anesthesia n = 21	Vaginal delivery without epidural anesthesia n = 147	p-value
Age (mean (SD))	33.00 (4.73)	34.59 (4.07)	32.52 (4.53)	32.60 (4.87)	0.043
Parity (mean (SD))	0.68 (0.81)	1.05 (0.94)	0.57 (0.75)	0.59 (0.76)	0.004
Pre-pregnancy body weight (kg) (mean (SD))	49.49 (16.79)	46.19 (18.44)	44.27 (22.66)	51.23 (15.06)	0.07
Duration of labor (min)					
1st stage (mean (SD))	499.01 (384.79)	475.63 (348.68)^†^	926.73 (738.98)	438.62 (220.87)	0.001
2nd stage (mean (SD))	60.97 (95.52)	51.22 (61.10)^†^	114.36 (297.81)	92.71 (79.29)	0.026
Outcome of delivery (%)					<0.001
Natural (n (%))	133 (62.7)	0	11 (52.4)	122 (83.0)	
Instrumental (n (%))	35 (16.5)	0	10 (47.6)	25 (17.0)	
Vacuum (n (%))	33 (15.6)	0	10 (47.6)	23 (15.6)	
Forceps (n (%))	2 (0.94)	0	0	2 (0.94)	
Cesarean section (n (%))	44 (20.8)	44 (100.0)	0	0	
Emergency cesarean section (n (%))	14 (6.6)	14 (/44=31.8)	0	0	
With epidural in labor (n (%))	2 (0.94)	2 (/44=4.5)	0	0	
Without epidural in labor (n (%))	9 (4.3)	9 (/44=20.5)	0	0	
Not in labor (n (%))	3 (1.4)	3 (/44=6.8)	0	0	
Perineal laceration					<0.001
None (n (%))	99 (46.7)	44 (100.0)	2 (9.5)	53 (36.1)	
1st (n (%))	11 (5.2)	0	0	11 (7.5)	
2nd (n (%))	98 (46.2)	0	19 (90.5)	79 (53.7)	
3rd (n (%))	4 (1.9)	0	0	4 (2.7)	
Neonatal weight (mean (SD))	2988.87 (364.89)	2949.82 (427.61)	3027.14 (346.34)	2995.09 (348.47)	0.68

The outcomes of delivery were spontaneous for 133 (62.7%) women, instrumental for 35 (16.5%), and cesarean section for 44 (20.8%). The degree of perineal laceration included none for 99 (46.7%) women, a first degree for 11 (5.2%), a second degree for 98 (46.2%), and a third degree for 4 (1.9%).

Among 212 postpartum women, 156 (73.6%) presented with PFD symptoms. Broken down into three groups, 59 women had POPDI (27.8%), 111 had CRADI (52.4%), and 114 had UDI (53.8%). The most prevalent PFD symptom was UDI in 114 (53.8%) women, of whom 79 (37.3%) experienced urine leakage related to increased abdominal pressure. The prevalence of PFD-20 items is shown in Supplementary Material Data 2.

The prevalence of PFD symptoms was statistically significant (p = 0.037) for all groups: cesarean section group, 61.4% (n = 27); epidural group, 90.5% (n = 19); and non-epidural group, 74.8% (n = 110) (Table 2).

**Table 2:** Prevalence of each section of the PFDI-20 symptoms among the three groups PFDI-20: Pelvic Floor Distress Inventory-20; POPDI: pelvic organ prolapse distress inventory; CRADI: colorectal anal distress inventory; UDI: urinary distress inventory

PFDI	Cesarean section	Vaginal delivery with epidural anesthesia	Vaginal delivery without epidural anesthesia	p-value
Overall (n = 212)	n = 44 (%)	n = 21 (%)	n = 147 (%)	
PFDI-20	27 (61.4)	19 (90.5)	110 (74.8)	0.037
POPDI	10 (22.7)	8 (38.1)	41 (27.9)	0.433
CRADI	18 (40.9)	15 (71.4)	78 (53.1)	0.067
UDI	19 (43.2)	14 (66.7)	81 (55.1)	0.174

Multivariable regression for pelvic floor symptom burden score showed the following results: the epidural group had an 8.67-point higher symptom burden score (estimate, 8.67 [0.03 to 17.3]) and the cesarean section group had a 7.61-point lower score (estimate, −7.61 [−14.2 to −1.03]), as compared to the non-epidural group, regarding the association between PFD and delivery mode (Table 3).

**Table 3:** Multivariate regression analysis of disease burden scores (PFDI-20, each section) PFDI-20: Pelvic Floor Distress Inventory-20; POPDI: pelvic floor disorders distress inventory; CRADI: colorectal anal distress inventory; UDI: urinary distress inventory.

	PFDI-20		POPDI		CRADI		UDI	
	Coefficient	95%CI	Coefficient	95%CI	Coefficient	95%CI	Coefficient	95%CI
Age	0.42	−0.13 to 0.98	0.13	−0.080 to 0.33	0.14	−0.096 to 0.37	0.16	−0.15 to 0.46
Parity	0.72	−2.54 to 3.99	0.15	−1.07 to 1.36	0.081	−1.29 to 1.45	0.50	−1.30 to 2.29
Pre-pregnancy body weight	−0.042	−0.20 to 0.11	−0.046	−0.10 to 0.011	−0.022	−0.086 to 0.043	0.025	−0.059 to 0.11
Exposure								
Vaginal delivery without epidural anesthesia	Reference		Reference		Reference		Reference	
Cesarean section	−7.61	−14.19 to −1.03	−1.20	−3.65 to 1.26	−2.29	−5.05 to 0.47	−4.12	−7.74 to −0.52
Vaginal delivery with epidural anesthesia	8.67	0.034~17.30	4.32	1.10~7.53	3.39	−0.23 to 7.01	0.96	−3.78 to 5.69

## Discussion

This study showed the prevalence of PFD symptoms in Japanese women in a large, single, rural center. It was relatively high, affecting 156 (73.6%) of the 212 women surveyed. The affected women were divided into three groups: 27.8% were affected by POPDI, 52.4% by CRADI, and 53.8% by UDI. The most prevalent PFD symptom was UDI in 114 (53.8%) women, of whom 79 (37.3%) experienced urine leakage associated with increased abdominal pressure. In the cesarean section group (n=44), PFD symptoms were prevalent in 27 (64.1%) women, including primipara and multipara, suggesting a high prevalence of PFD symptoms in the cesarean section group (Table 2).

A previous study reported that higher scores were recorded on the PFDI-20 (p=0.003) questionnaire in late pregnancy and the early postpartum period rather than in early pregnancy; hence, postpartum PFD is usually associated with delivery as a risk factor [16].

Limited data are available on the prevalence of PFD symptoms in Japanese women; hence, it is important to assess their prevalence.

PFD is widespread in the immediate postpartum period; it tends to resolve six months later; however, it persists in a considerable number of cases [17]. It is remarkable that our results show high rates of prevalence of PFD symptoms even 6-15 months postpartum, as their prevalence in other countries is relatively variable. In Australia, PFDI prevalence was seen in 97% (n = 196) of the patients who answered the questionnaire, with 9% of them affected by POPDI, 79% by CRADI, and 82% by UDI [2]. Conversely, a study on Israeli women showed that 12.8% of surveyed women were affected by POPDI, 20.7% by CRADI, and 15.8% by UDI at three months postpartum [3].

Our results regarding the prevalence of PFD symptoms in Japanese women contradicted those of previous studies [2,3]. We hypothesized several reasons for this. The first reason could be due to the differences in modes of delivery. The Australian study only enrolled patients who delivered vaginally, excluding those who underwent cesarean sections, which would explain the relatively higher prevalence of PFD in their patients. Our study demonstrated a lower prevalence of PFD symptoms with cesarean section than with vaginal delivery. The second reason involves the timing of the study period. Women at three months postpartum may still be less active in their daily routines and may be bearing lesser loads on their bodies due to the lighter body weight of their infants, thereby exerting a lesser burden on their pelvic floor muscles. This would explain the lower prevalence of PFD in the Israeli study [3].

Furthermore, our result would have a higher prevalence than other studies due to our methodology, wherein any response to the questionnaire, even the lowest possible score of 1, would be considered indicative of a positive symptom. We believe that individuals who are currently presenting symptoms, regardless of the severity of their scores, may still be at risk for the development of their symptoms in the future; thus, our result would be significant in this aspect [18].

To date, no prominent factor has been identified for these differences. However, these differences could arise from the type of perinatal management and the timing of the study. Therefore, more studies exploring these factors should be conducted in the future.

Regarding the association between PFD and the use of epidural anesthesia during labor, a higher disease burden score of 8.67 points (estimate: 8.67 [0.03 to 17.3]) was observed in the epidural group compared with the non-epidural group (Table 3).

Sartore et al. concluded that the use of epidural anesthesia is not associated with symptoms related to perineal trauma and pelvic floor muscle weakness [19]. We adjusted for the anesthetic method used and the duration of labor during analysis, but the results remained unchanged; the epidural group still showed a higher risk for PFD. The measurement tools and methodologies used for assessing pelvic floor function in Sartore’s study were dissimilar from those used in this study, explaining the variations between the two studies.

Epidural anesthesia is related to a prolonged second stage of labor [4]. Vaginal delivery with epidural anesthesia is associated with a higher risk of instrumental vaginal deliveries compared with labor without epidural anesthesia [5]. Therefore, epidural anesthesia is believed to harm pelvic floor function. The results of these studies are not contradictory to ours.

Interpreting the association between PFD and delivery mode, a lower disease burden score (estimate, −7.61 (−14.2 to −1.03)) was observed in the cesarean group compared with the non-epidural group (Table 3). This indicates that, in the short term, cesarean sections are associated with a lower incidence of PFD in postpartum women compared with vaginal delivery, whether non-epidural or epidural. Furthermore, Baud et al. revealed that women were more likely to report urinary incontinence after vaginal delivery than after cesarean sections at six years postpartum. In contrast, women more frequently reported symptoms of sexual dysfunction, especially dyspareunia, after cesarean sections than after vaginal deliveries. Therefore, the advantages, disadvantages, and long-term consequences of the delivery mode should always be considered [20].

Thus, based on the results of our study, we recommend that cesarean delivery not be chosen over vaginal-based delivery solely based on the lower risk of PFD associated with cesarean deliveries.

Our study demonstrated a high prevalence of PFD symptoms in women at six to fifteen months postpartum. Therefore, an accurate diagnosis of PFD in postpartum women, along with appropriate and regular follow-up until improvement of their symptoms, is crucial.

Regular pelvic floor muscle training in postpartum women (n=50) showed significant improvements in pelvic floor function as per the PFDI-20 (mean change of −41.8; p<0.001) [21]. Therefore, healthcare workers should consider early identification of symptoms to provide care and thereby reduce PFD development. This would be more crucial in the epidural group, which has a higher prevalence of PFD symptoms. A study reported that in women with relatively mild pelvic floor symptoms, an improvement of 13.5 points in the PFDI-20 score can be considered clinically relevant [22]. This minimal yet significant change can be used for clinical trial planning and evaluation of treatment or intervention effects. Hence, our result of 8.67 points could be considered a clinical risk. Healthcare workers should therefore inform pregnant women of the possible increase in the risk of pelvic floor disorders with anesthesia.

This study has several limitations. First, the PFD-20 is an evaluation of symptoms, not of pathology. Hence, even though the prevalence of UDI was high, it cannot be equated with the severity of stress urinary incontinence. Second, this study was based on data from a single center; therefore, the composition of the catchment population and resources may potentially limit the generalizability of our results in Japan. Third, a low response rate of 67.7% raised suspicions that non-responders probably had no or few PFD symptoms. Fourth, regarding analytic limitations, we considered instrument delivery and perineal laceration as mediators; we could not separate the effect of these mediators from the methods of delivery. Furthermore, factors that affect the prognosis of PFD, such as episiotomy, birth weight, and fetal biparietal diameter, were considered predictors, not confounders. However, we believe that this did not affect the study's results. Additionally, since this was an observational study, we were not able to adjust for unmeasured confounders. Therefore, the effect of epidurals on PFDI may have been biased. For example, the estimated fetal body weight could have affected the implementation of both epidurals and PFDI, which could not be investigated in this study. More factors need to be prospectively investigated in future studies.

## Conclusions

In conclusion, the results of this study suggest that pelvic floor disorders are highly prevalent in Japanese women after giving birth. The use of the PFDI-20 questionnaire has facilitated the assessment of these conditions and revealed urinary distress as the most common symptom among postpartum women. Additionally, the study found an association between epidural anesthesia and a higher disease burden score, indicating a potential role for this intervention in the development of pelvic floor disorders.

## Figures and Tables

**Figure 2:**
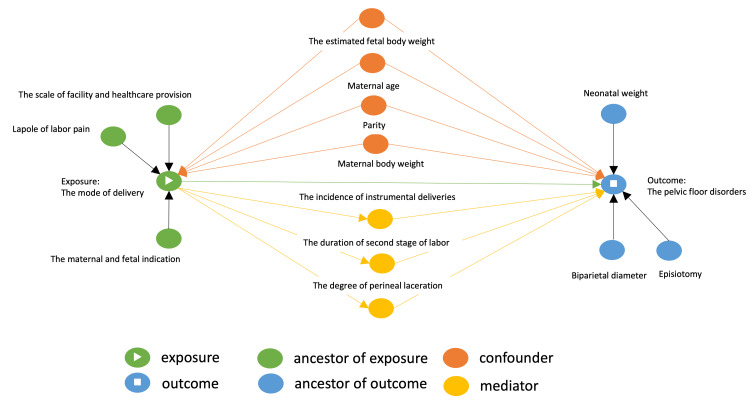
The causal directed acyclic graphs (DAGs)

## Appendices

Supplementary Material data 1: The causal directed acyclic graphs (DAGs)

Supplementary material data 2: The prevalence of each PFDI-20 question

**Table 4:** The prevalence of each PFDI-20 question PFDI-20: Pelvic Floor Distress Inventory-20; POPDI: pelvic floor disorders distress inventory; CRADI: colorectal anal distress inventory; UDI: urinary distress inventory.

PFDI	Cesarean section	Vaginal delivery with epidural anesthesia	Vaginal delivery without epidural anesthesia	p-value
n (%)	44 (%)	21 (%)	147 (%)	
POPDI_Q1	3 (6.8)	3 (14.3)	8 (5.4)	0.311
POPDI_Q2	3 (6.8)	4 (19.0)	20 (13.6)	0.326
POPDI_Q3	2 (4.5)	3 (14.3)	6 (4.1)	0.140
POPDI_Q4	3 (6.8)	3 (14.3)	7 (4.8)	0.230
POPDI_Q5	3 (6.8)	2 (9.5)	14 (9.5)	0.855
POPDI_Q6	0 (0.0)	1 (4.8)	0 (0.0)	0.010
CRADI_Q7	4 (9.1)	6 (28.6)	25 (17.0)	0.135
CRADI_Q8	7 (15.9)	9 (42.9)	33 (22.4)	0.052
CRADI_Q9	0 (0.0)	0 (0.0)	3 (2.0)	0.510
CRADI_Q10	0 (0.0)	0 (0.0)	7 (4.8)	0.202
CRADI_Q11	8 (18.2)	5 (23.8)	30 (20.4)	0.868
CRADI_Q12	4 (9.1)	5 (23.8)	13 (8.8)	0.104
CRADI_Q13	7 (15.9)	3 (14.3)	27 (18.4)	0.859
CRADI_Q14	3 (6.8)	3 (14.3)	7 (4.8)	0.230
UDI_Q15	3 (6.8)	7 (33.3)	21 (14.3)	0.018
UDI_Q16	2 (4.5)	1 (4.8)	13 (8.8)	0.561
UDI_Q17	8 (18.2)	9 (42.9)	62 (42.2)	0.013
UDI_Q18	6 (13.6)	2 (9.5)	29 (19.7)	0.389
UDI_Q19	3 (6.8)	0 (0.0)	2 (1.4)	0.084
UDI_Q20	6 (13.6)	2 (9.5)	12 (8.2)	0.552
